# Glo-In-One-v2: holistic identification of glomerular cells, tissues, and lesions in human and mouse histopathology

**DOI:** 10.1117/1.JMI.12.6.061406

**Published:** 2025-07-28

**Authors:** Lining Yu, Mengmeng Yin, Ruining Deng, Quan Liu, Tianyuan Yao, Can Cui, Junlin Guo, Yu Wang, Yaohong Wang, Shilin Zhao, Haichun Yang, Yuankai Huo

**Affiliations:** a Vanderbilt University, Department of Computer Science, Nashville, Tennessee, United States; b Vanderbilt University Medical Center, Department of Pathology, Microbiology and Immunology, Nashville, Tennessee, United States; c UT MD Anderson Cancer Center, Department of Anatomical Pathology, Houston, Texas, United States; d Vanderbilt University Medical Center, Department of Biostatistics, Nashville, Tennessee, United States

**Keywords:** open-source, renal pathology, glomerular segmentation, whole-slide image, glomerular lesion, transfer learning

## Abstract

**Purpose:**

Segmenting intraglomerular tissue and glomerular lesions traditionally depends on detailed morphological evaluations by expert nephropathologists, a labor-intensive process susceptible to interobserver variability. Our group previously developed the Glo-In-One toolkit for integrated glomerulus detection and segmentation. We leverage the Glo-In-One toolkit to version 2 (Glo-In-One-v2), which adds fine-grained segmentation capabilities. We curated 14 distinct labels spanning tissue regions, cells, and lesions across 23,529 annotated glomeruli from human and mouse histopathology data. To our knowledge, this dataset is among the largest of its kind to date.

**Approach:**

We present a single dynamic-head deep learning architecture for segmenting 14 classes within partially labeled images from human and mouse kidney pathology. The model was trained on data derived from 368 annotated kidney whole-slide images with five key intraglomerular tissue types and nine glomerular lesion types.

**Results:**

The glomerulus segmentation model achieved a decent performance compared with baselines and achieved a 76.5% average Dice similarity coefficient. In addition, transfer learning from rodent to human for the glomerular lesion segmentation model has enhanced the average segmentation accuracy across different types of lesions by more than 3%, as measured by Dice scores.

**Conclusions:**

We introduce a convolutional neural network for multiclass segmentation of intraglomerular tissue and lesions. The Glo-In-One-v2 model and pretrained weight are publicly available at https://github.com/hrlblab/Glo-In-One_v2.

## 1 Introduction

Whole-slide imaging (WSI) provides high-resolution views of tissue, significantly advancing quantitative analysis in nephropathology—particularly in glomerular evaluation.^1^ Within renal pathology, a field known for its complexity in image interpretation, glomeruli are essential functional units for clinical assessment.^2^ To automate glomerular detection and segmentation, our team previously developed the Glo-In-One toolkit,^3^ based on convolutional neural networks (CNNs). Although effective, the original Glo-In-One was limited to whole-tuft segmentation and lacked the ability to capture sub-glomerular structures.

**Fig. 1:**
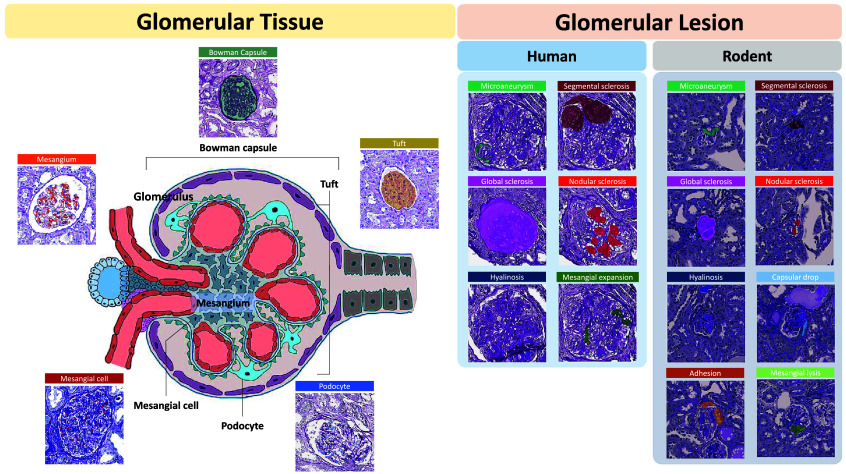
This figure presents fine-grained classes of intraglomerular tissue, including Bowman’s capsule (Cap), tuft (Tuft), mesangium (Mes), mesangial cells (Mec), and podocytes (Pod). It also highlights the glomerular lesions observed in rodents and humans: AH, adhesion; CD, capsular drop; GS, global sclerosis; HS, hyalinosis; ML, mesangial lysis; MA, microaneurysm; NS, nodular sclerosis; ME, mesangial expansion; SS segmental sclerosis.

In this study, we introduce Glo-In-One-v2, an enhanced toolkit that supports fine-grained glomerular lesion segmentation. It applies 14 labels spanning tissue regions, cells, and lesions across a dataset of 23,529 annotated glomeruli—one of the largest of its kind. The corresponding labels are illustrated in Fig. 1. Glo-In-One-v2 employs a dynamic deep learning architecture to segment 14 classes in partially labeled human and mouse pathology images.

Although many recent studies have explored deep learning for glomerular quantification,^4^^–^^8^ few have focused specifically on glomerular lesions. These lesions are key indicators of kidney damage and are closely associated with a range of renal diseases.^9^ However, their small size and interobserver variability make them more difficult to segment than healthy glomeruli. Manual annotation is time-consuming, inconsistent, and requires expert input, limiting scalability and reproducibility. To improve lesion segmentation, we incorporate a cross-species dataset that includes rodent kidney samples. Rodents, particularly mice, are widely used in preclinical nephrology research due to their genetic similarity to humans, short lifespans, and suitability for experimental manipulation.^10^^,^^11^ Importantly, rodent models play a central role in translational research, bridging basic science and clinical application in drug development, diagnostics, and therapeutic evaluation.^12^^–^^14^ By leveraging structural and pathological similarities between rodent and human glomeruli, our model learns transferable features that enable accurate segmentation of human samples, even when trained partially on rodent data.

Our contribution is threefold:

• We evaluate the feasibility of cross-species transfer learning to overcome the key limitation of limited availability and coverage of human glomerular lesion segmentation data, by leveraging corresponding lesion data from rodents. To the best of our knowledge, this is the first study to apply transfer learning across species for the critical task of glomerular lesion segmentation.
• We present Glo-In-One v2, a framework that delivers fully automated segmentation of intraglomerular structures and fine-grained glomerular lesions, enabling more comprehensive pathological characterization across diverse disease populations. This advancement is made possible by a newly curated dataset comprising over 23,000 glomerular image patches from 368 WSIs, each annotated with five intraglomerular tissue types and nine lesion categories.
• Beyond fine-grained lesion segmentation, Glo-In-One v2 supports an end-to-end pipeline encompassing glomerulus detection, segmentation, and lesion quantification directly from WSIs. To support clinical users with minimal programming experience, the entire pipeline is packaged as a containerized, open-source Docker toolkit. This allows users to quantify all glomeruli in a given WSI using a single command line. The toolkit is publicly available at https://github.com/hrlblab/Glo-In-One_v2.

## 2 Related Work

### 2.1 Glomeruli Segmentation

Several studies have explored the segmentation of renal structures beyond the glomerulus, including the tubules, blood vessels, and interstitial regions.^15^^,^^16^ In addition, other works have focused on intraglomerular components such as Bowman’s capsule, the glomerular tuft, and the mesangium.^17^^–^^19^ Accurate segmentation of both glomeruli and their internal substructures provides valuable insight into kidney disease, supporting the classification of pathological findings and enabling the development of prognostic models through quantitative analysis of histopathological regions. However, the diversity of intraglomerular tissue classes addressed in current research remains limited, suggesting room for further expansion.

Moreover, most existing studies on glomerular analysis rely on patch-wise approaches. These include the detection^20^^,^^21^ and segmentation^17^ of multiple glomeruli within large image patches or binary classification tasks on smaller image regions.^22^ Although some efforts have attempted to classify or identify glomerular lesions, these tasks require models to capture more complex and subtle histological features to differentiate among various lesion types effectively.

Recent work by Nan et al.^23^ proposed methods for detailed recognition of glomerular lesions from WSIs, targeting both segmentation and classification. Similarly, the analytic renal pathology system^18^ and the work by Akatsuka and Horai^24^ have advanced automated lesion and cell-type identification using deep learning. However, these studies often address a limited set of lesion types, potentially omitting clinically relevant features and underrepresenting the complexity and variability of glomerular pathology. In addition, segmentation tasks present a higher degree of difficulty than classification, as they require a deeper understanding of both structural context and spatial detail within histological images.

Building on this foundation, our study introduces a dataset that encompasses five intraglomerular tissue classes and nine glomerular lesion types, offering broader coverage than prior works. This expanded label space enables the development of models that are better equipped to differentiate among subtle yet clinically significant lesion patterns, improving the granularity and clinical relevance of automated kidney pathology analysis.

### 2.2 Rodent-to-Human Transfer Learning

Traditional transfer learning approaches for domain adaptation—such as rodent-to-human segmentation—often adopt a zero-shot setting, where a model trained solely on rodent data is directly applied to human test data. However, due to inherent distributional differences among species, particularly in histopathological features, generalizing across domains—especially for tasks such as glomerular lesion segmentation—remains challenging.

Furthermore, existing segmentation networks have demonstrated notable performance in various medical imaging tasks. Ronneberger et al.^25^ proposed U-Net, a symmetric encoder–decoder network with skip connections that enables accurate biomedical image segmentation. González et al.^26^ proposed a multi-structure segmentation method using partially labeled data to address the challenge of incomplete annotations. Lutnick et al.^27^ implemented DeepLab v2 to detect sclerotic glomeruli as well as regions of interstitial fibrosis and tubular atrophy. Strudel et al.^28^ proposed Segmenter that extends vision transformer (ViT) to capture global context from the earliest layers. Hatamizadeh et al.^29^ proposed Swin UNETR combining a hierarchical Swin Transformer encoder with a CNN-based decoder to improve the performance of modeling long-range information. However, most of the recent works handle multi-label segmentation by allocating separate output channels for each class. Although this design allows per-class prediction, it overlooks potential inter-class relationships and shared contextual information that could impact segmentation accuracy, particularly in complex tissue structures.

**Fig. 2:**
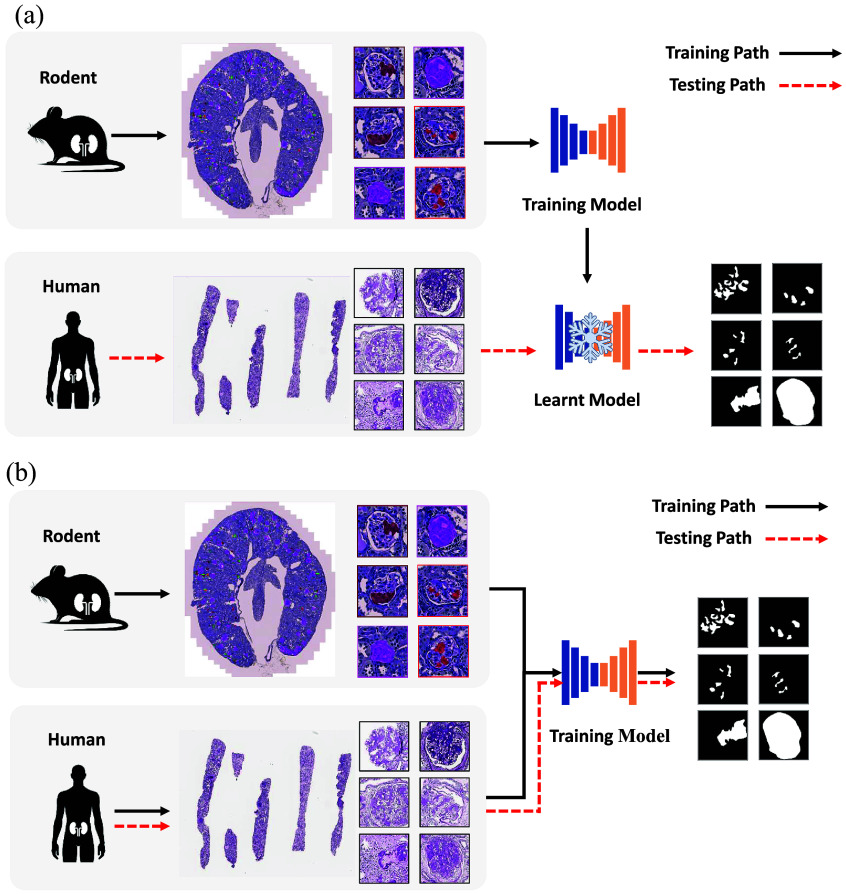
This figure provides an overview of transfer learning for glomerular segmentation from rodent to human, where panel (a) illustrates the direct adaptation of a model trained on rodent data to human tasks without incorporating any knowledge from the human domain. In contrast, panel (b) demonstrates the use of a model that integrates knowledge learned from both rodent and human data. In the figures, the black arrows represent training paths, whereas the red arrows indicate testing paths. (a) Rodent-to-human zero-shot transfer learning. (b) Rodent-to-human hybrid transfer learning.

Thus, we introduce a multi-label segmentation network specifically optimized for the dataset to improve predictive performance. In contrast to traditional zero-shot rodent-to-human transfer learning strategies, our approach utilizes rodent data as an auxiliary source of support (Fig. 2). This design allows the network to better generalize and predict human glomerular lesions by transferring representational knowledge learned during training on rodent samples.

## 3 Method

### 3.1 Segmentation Network

**Fig. 3:**
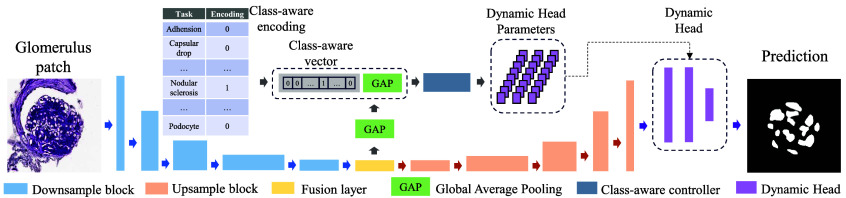
This figure illustrates the proposed network architecture, which comprises a residual U-Net backbone, a class-aware controller, and a single dynamic segmentation head. A class-aware knowledge encoder is integrated into the framework for multi-label segmentation, and a feature-based fusion block is employed to aggregate the features into the final dynamic head parameters.

We present a single segmentation network inspired by the work of Deng et al.,^30^ which leverages a residual U-Net backbone to segment various glomerular classes from partially labeled pathology images. The network is specifically tailored for our intraglomerular tissue and lesion segmentation tasks. As illustrated in Fig. 3, given a patch image, the backbone employs downsampling blocks to progressively extract high-level semantic features, and upsampling blocks to gradually restore spatial resolution. On top of this backbone, the architecture integrates a class-aware controller that fuses class-specific knowledge with image features, followed by a dynamic head for task-specific segmentation.

Unlike fully labeled datasets, each image included in a partially labeled dataset contains the annotations of only a specific class involved. To enable task awareness, we treat each segmentation of a specific tissue or lesion class as a distinct task, encoding it using an m-dimensional one-hot vector.^31^ The m is the number of classes. The encoding calculation for $T_{k}$, a class-aware vector of the i’th class of lesion is shown as follows:

$$T_{k}=\{\begin{array}{ll} 1, & \text{if}\;k=i\\ 0, & \text{otherwise}, \end{array}\;k=1,2,\ldots ,m.$$ (1)

Dynamic filter generation^32^ was introduced to generate the kernels specialized to a particular class of segment tasks. A class-aware controller is used to aggregate the image feature F by combining a global average pooling with $T_{k}$. The kernel parameters ω are computed as follows:

$$\omega =\phi (\mathrm{GAP}(F)∥T_{k};\Theta_{\phi }),$$ (2)

where $\Theta_{\phi }$ represents the controller parameters, “∥” represents the concatenation operation to combine high-level image features and the class-aware vector, and ϕ reprensents a task-specific controller with a single two-dimensional convolutional layer.

The dynamic head was designed to achieve multi-label segmentation with three layers, denoted by $\omega_{1}$, $\omega_{2}$, and $\omega_{3}$. The predictions of lesions can be generated as follows: $P=M*\omega_{1}*\omega_{2}*\omega_{3}$ where * is the convolution, and M is the output from the decoder.

### 3.2 Glomerular Mining with Glo-In-One

To obtain a large-scale dataset of unannotated glomerular images, we leverage our previous work, Glo-In-One, which enabled the collection of over 30,000 such images through extensive web-based image mining. This process involved the separation of compound figures retrieved via the National Institutes of Health (NIH) Open-i^®^ search engine. Further details of the compound figure collection and image mining methodology can be found in Ref. 33.

### 3.3 Containerization

**Fig. 4:**
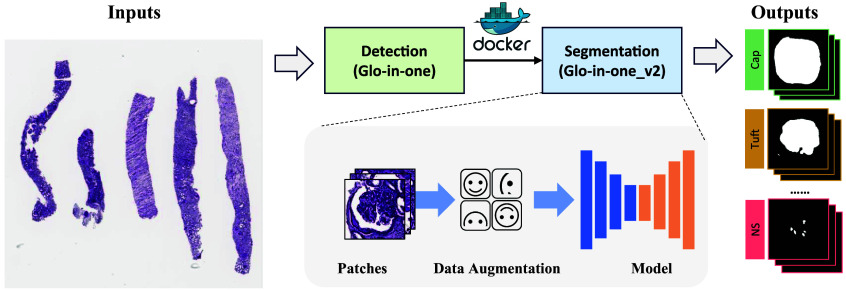
This figure provides an overview of the Glo-In-One-v2 toolkit. The proposed toolkit is able to achieve 14 segmentation classes using a single Docker command line. The input consists of raw WSIs, and the output is a holistic segmentation of the glomeruli. The detection module, inherited from the previous toolkit version, delivers quantitative detection of glomeruli. The segmentation module utilizes a trained model, developed from patches extracted from WSIs with manual annotations provided by medical experts.

To facilitate glomerular quantification for non-technical users, we developed the Glo-In-Onev2 toolkit; as shown in Fig. 4, an all-in-one solution that enables comprehensive glomerular detection and segmentation through a single, user-friendly command. By containerizing both the detection and segmentation modules within a Docker environment, we streamline the process so that users only need to input WSIs to obtain sophisticated multi-channel segmentation masks as output. Each channel in the mask is mapped to a specific intraglomerular tissue type or glomerular lesion class, offering a granular view tailored for in-depth analysis. This approach not only eliminates the need for specialized technical skills but also significantly reduces the time and effort required for advanced glomerular analysis, making it accessible and practical for a wider range of users, from clinical practitioners to researchers.

### 3.4 Relationship with Previous Version

Both Glo-In-One and Glo-In-One v2 are designed for glomerular detection and segmentation. In the former, glomerular regions are first identified and then segmented using a DeepLab v3 network. However, segmentation in the original version is limited to glomerular boundaries only.

Glo-In-One v2 builds upon this foundation by retaining the original glomerular mining and detection strategies while introducing a more powerful single-network multi-label dynamic segmentation framework. This upgraded architecture enables finer and more accurate segmentation performance. Crucially, the new version extends beyond simple glomerular delineation to support detailed segmentation of intraglomerular tissue types and glomerular lesions, thereby providing more comprehensive insights into renal histopathology.

For the entire pipeline, we integrate the functionalities of both the original Glo-In-One and Glo-In-One v2 and package them into a Docker container. The pipeline takes WSIs as input, performs glomerular detection using the original version, and conducts segmentation using the updated v2, enabling a fully automated, end-to-end processing workflow.

## 4 Experiments and Results

### 4.1 Data Collection

In this study, we curated a dataset comprising over 23,529 annotated glomerular patches obtained from 368 WSIs of renal pathology. Of these, as detailed in Table 1, 16,943 patches were manually annotated by renal pathologists, whereas 6586 patches were derived from the Kidney Precision Medicine Project (KPMP) spatial segmentation dataset.^34^ The annotations encompass intraglomerular tissue (Cap, Tuft, and Mes), cellular components (Pod and Mec), and a variety of glomerular lesions (AH, CD, GS, HS, ME, ML, MA, NS, and SS). All patches were extracted at the original highest scanning resolution of the WSIs and subsequently cropped and resized to 512×512  pixels.

**Table 1:** Distribution of subclasses in glomeruli.

	Region			Cell		Lesion									Total
	Cap	Tuft	Mes	Prod	Mec	AH	CD	GS	HS	ME	ML	MA	NS	SS	
Rodent	1393	5542	5542	1157	789	85	62	380	196	—	71	203	342	196	
Human	6586	—	—	—	—	—	—	369	35	227	-	56	229	69	
Quantity	7979	5542	5542	1157	789	85	62	749	231	227	71	259	571	265	23,529

The dataset was stratified into training, validation, and test sets in a 6:1:3 ratio across all classes, ensuring patient-level splits to prevent data leakage (detailed split result shown in Table 2).

**Table 2:** Training, validation, and testing sets.

	Train	Val	Test
Intraglomerular tissue	12,410	2393	6206
Human lesion	513	200	272
Rodent lesion	969	159	407

### 4.2 Experimental Design

Utilizing an image pool concept inspired by Cycle-GAN,^35^ we fed images into the model in batches of four, with the image pool size set matching the number of involved classes. When the number of images in the pool surpasses the batch size, a batch of images is selected for model input.

For the holistic segmentation task, the model was trained on the complete training set, encompassing all annotated glomerular tissue and lesion types. In the rodent-to-human transfer learning task, we leveraged a model trained on both rodent and human data to make independent predictions on mouse and human test sets. In addition, to evaluate the models’ susceptibility to label interference, we introduced a tissue label into the training set. These added labels exhibit overlapping or superset relationships with the target lesion labels in the test set, allowing us to assess the models’ robustness. For comparison, we evaluated four different training strategies: (1) human-to-human (H2H)—the model was trained on the human lesion training set and evaluated on the human lesion test set; (2) rodent-to-human (R2H)—the model was trained on the rodent lesion training set and evaluated on the human lesion test set; (3) rodent-and-human to human (R&H2H)—the model was trained on a combined lesion dataset from both rodent and human samples and evaluated on the human lesion test set; and (4) rodent-and-human to human (R&H2H+T)—the model was trained on a combined lesion and tissue dataset from both rodent and human samples and evaluated on the human lesion test set.

Model performance was evaluated using the Dice similarity coefficient (Dice). The best-performing model was selected based on the highest average Dice score on the validation set across 200 training epochs, and this model was used for final evaluation. All experiments were conducted on an NVIDIA RTX A5000 GPU with 24 GB of VRAM.

We compared the introduced network to baseline models, including (1) multiple individual U-Net models (U-Nets),^25^ (2) multiple individual DeepLabv3 models (DeepLabv3s),^27^ and (3) a multi-class segmentation model for partially labeled datasets^26^ for renal pathology quantification. In addition, the performance of the network was evaluated against transformer baselines (4) a hybrid neural network architecture that combines the Swin Transformer with the U-Net transformer encoder for enhanced medical image segmentation (Swin UNETR),^29^ and (5) an approach to semantic segmentation based on the Vision Transformer (Segmenter).^28^ All of the parameter settings are followed by the original paper.

### 4.3 Results

#### 4.3.1 Holistic segmentation

**Fig. 5:**
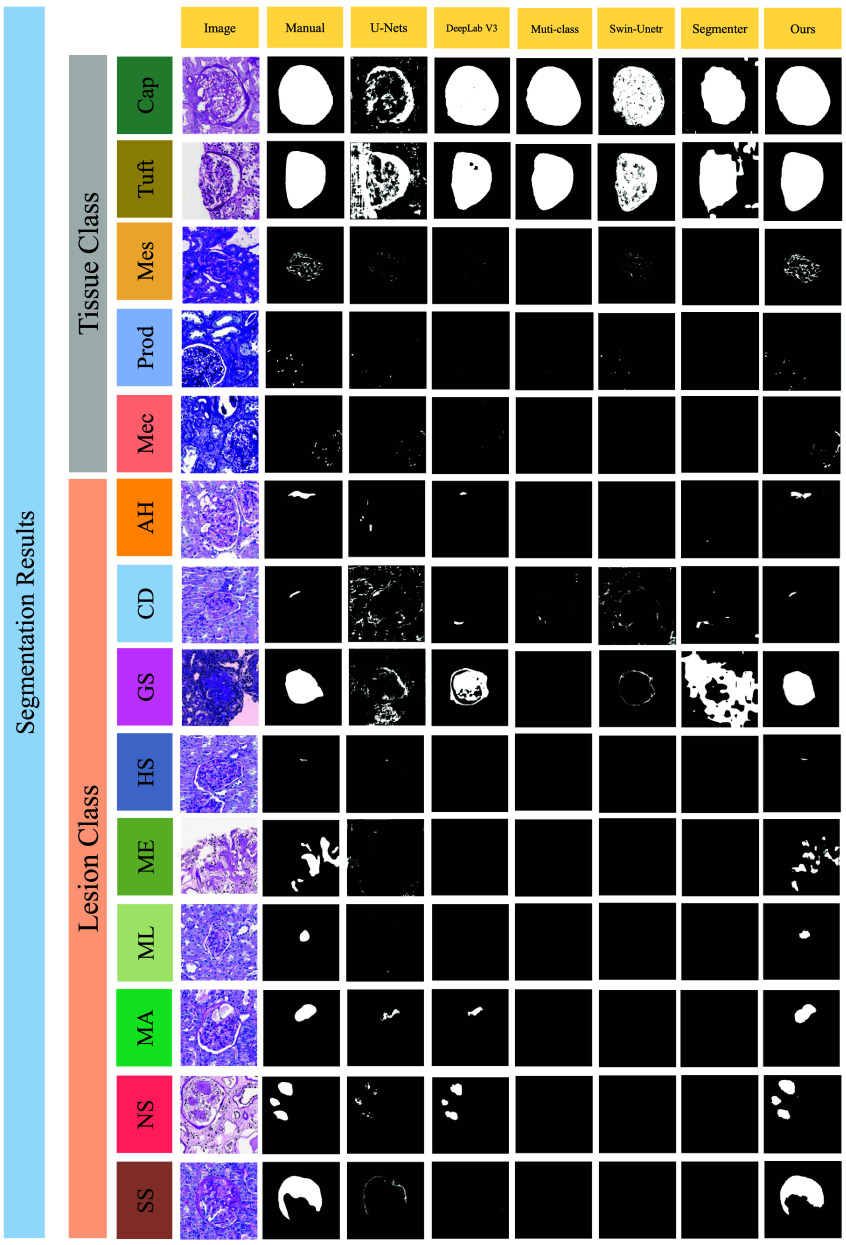
This figure displays the qualitative outcomes of various segmentation methods for all classes of glomeruli. The first column features the original, unannotated images, whereas the second column shows the manual segmentation results.

Table 3 shows the performance metrics for the segmentation of each class of glomerular tissue and lesion over the entire rodent and human dataset. Figure 5 presents the qualitative results about the performance of different methods on the multi-label dataset. The experimental results show that our trained model as a single multi-label model can achieve better performance, achieving an average Dice score of 76.5%, on prediction classes of both glomerular tissues and lesions than baseline methods, such as CNN-based (i.e., DeepLabV3) and transformer-based (i.e., Swin UNETR).

**Table 3:** Performance of different models for glomerular tissue and lesion segmentation. Dice similarity coefficient (%; the higher, the better) is used for evaluation. The bold mark indicates the best performance.

Method	Backbone	Region			Cell		Lesion		
		Cap	Tuft	Mes	Prod	Mec	AH	CD	GS
U-Nets^25^	CNN	74.6	59.4	59.8	71.1	60.9	50.2	50.8	46.0
DeepLabV3^27^	CNN	82.9	67.3	51.8	58.4	50.9	50.2	58.7	71.1
Multi-class^26^	CNN	95.0	46.9	49.3	49.9	49.9	49.7	59.0	44.3
Swin UNETR^29^	Transformer	82.9	71.1	73.4	69.8	50.1	50.2	52.3	47.7
Segmenter^28^	Transformer	72.8	72.0	49.4	50.5	50.9	46.7	55.8	55.7
Ours	CNN	**96.3**	**97.0**	**89.5**	**76.4**	**66.6**	**59.6**	**67.5**	**93.6**
Method	Backbone	Lesion						Average	
		HS	ME	ML	MA	NS	SS		
U-Nets^25^	CNN	63.4	44.1	49.5	60.2	52.4	52.7	56.8	
DeepLabV3^27^	CNN	56.9	49.1	50.4	56.2	64.0	50.2	58.4	
Multi-class^26^	CNN	49.9	49.1	49.6	49.3	49.0	48.1	52.8	
Swin UNETR^29^	Transformer	50.3	49.1	49.6	51.3	49.5	48.2	56.8	
Segmenter^28^	Transformer	50.5	50.0	49.6	55.5	51.2	50.2	54.3	
Ours	CNN	**73.2**	**66.2**	**57.1**	**71.5**	**76.6**	**79.4**	**76.5**	

The results suggest that, although multi-head architectures face challenges in capturing spatial relationships among objects (e.g., subset-superset associations between the bowman capsule and tuft), the introduced dynamic-head approach outperforms other methods.

#### 4.3.2 Rodent-to-human transfer learning

As shown in Table 4, we evaluated the predictive accuracy of models trained solely on rodent samples across various common lesion categories in human subjects. In the human-to-human (H2H) experiments, the limited availability of human samples proved insufficient for the model to acquire the necessary knowledge to predict certain classes accurately. When we opted to apply rodent models directly to human prediction (R2H) in a zero-shot transfer learning manner, performance was suboptimal due to morphological differences and other domain gaps. However, using the hybrid transfer learning strategy (R&H2H), where rodent samples serve as an auxiliary source for human sample prediction, performance was enhanced, achieving an average Dice score of 70.4%, when our model is able to acquire knowledge more comprehensively from both human and rodent data by leveraging the decent performance on the rodent data, as detailed in Table 6 in the appendix.

**Table 4:** Performance of different models and strategies: human-to-human (H2H), rodent-to-human (R2H), rodent and human-to-human (R&H2H), and rodent and human-to-human plus additional tissue data (R&H2H+T). Dice similarity coefficient (%; the higher, the better) is used for evaluation. The bold mark indicates the best performance.

Method	Approach	Human glomerular lesion					Average
		GS	HS	MA	NS	SS	
U-Nets^25^	H2H	91.4	49.9	49.4	67.0	47.8	61.1
	R2H	70.6	49.9	50.9	67.8	47.8	57.4
	R&H2H	65.9	55.2	55.6	59.0	47.8	56.7
	R&H2H + T	39.7	51.0	52.9	53.2	52.9	49.9
DeepLabV3^27^	H2H	95.0	54.1	55.7	70.9	52.1	65.6
	R2H	78.2	52.7	49.9	56.4	60.8	59.6
	R&H2H	74.8	54.1	54.1	68.5	50.7	60.4
	R&H2H + T	79.3	52.7	51.3	60.6	47.8	58.3
Multi-class^26^	H2H	92.6	49.9	49.4	48.4	47.8	57.6
	R2H	78.0	49.9	49.4	48.4	47.8	54.7
	R&H2H	91.4	49.9	49.4	48.4	47.8	57.4
	R&H2H + T	41.0	50.0	49.4	48.3	47.8	47.3
Swin UNETR^29^	H2H	90.4	49.9	49.5	65.0	47.8	60.5
	R2H	81.5	50.1	49.5	53.3	49.6	56.8
	R&H2H	79.3	53.4	49.4	63.3	47.8	58.6
	R&H2H + T	44.1	49.9	49.4	48.4	47.8	47.9
Segmenter^28^	H2H	83.3	50.2	50.6	49.2	48.0	56.3
	R2H	82.5	50.4	50.7	49.4	49.4	56.5
	R&H2H	66.6	49.9	50.0	55.9	48.4	54.2
	R&H2H + T	61.6	49.9	49.4	50.4	47.2	51.7
Ours	H2H	94.3	49.9	50.5	**76.5**	68.5	67.9
	R2H	79.3	49.9	50.5	67.8	69.5	63.4
	R&H2H	94.0	**57.7**	55.3	75.3	69.6	70.4
	R&H2H + T	**95.2**	54.5	**58.7**	76.1	**71.3**	**71.2**

Moreover, despite potential disturbances from tissue classes with superset relationships (e.g., Bowman’s capsule tissue and glomerular lesions), our model demonstrated robust performance, achieving an average Dice score of 71.2%, effectively overcoming these challenges.

**Fig. 6:**
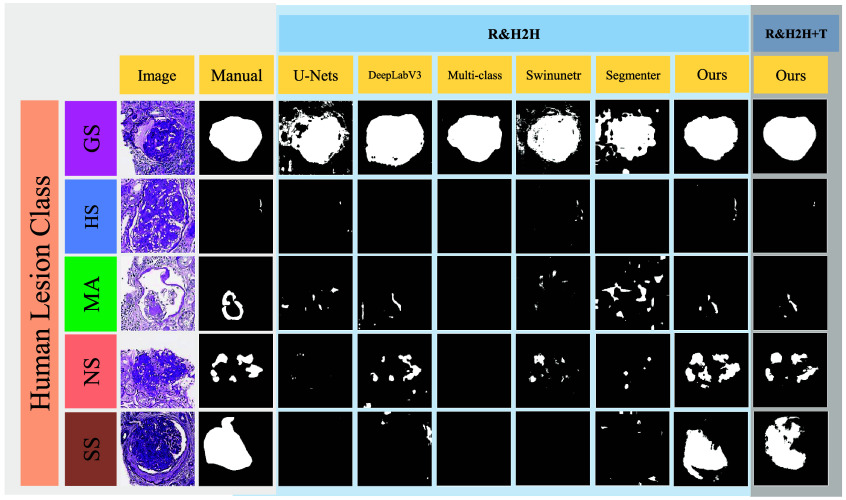
This figure displays the qualitative outcomes of various segmentation methods for all common classes of glomerular lesions. The first column features the original, unannotated images, whereas the second column shows the manual segmentation results.Subsequent columns belong to two section: “R&H2H” and “R&H2H + T.”

In addition, to evaluate the performance of models on human samples and compare the effect of transfer learning, Table 4 presents performance metrics for all lesion classes in the complete human dataset. Figure 6 provides a qualitative comparison of the performance of different methods in adapting rodent models for prediction tasks on the human dataset.

#### 4.3.3 Ablation study

Table 5 presents the performance using different species. In this setting, the data ratio between human and rodent is 1:5, simulating the scenario that human data is less than rodent data. The best performance was achieved with both our architecture and a combined dataset of human (H) and rodent (R) samples.

**Table 5:** Ablation studies on using data from different species.

	H	R	Human glomerular lesion					Average
			GS	HS	MA	NS	SS	
ResUNet	—	✓	68.8	52.6	50.4	56.7	59.2	57.5
ResUNet	✓	—	84.0	48.8	48.6	72.6	66.3	64.0
ResUNet	✓	✓	88.0	47.7	48.7	73.1	64.6	64.4
Glo-In-One v2 (ours)	—	✓	63.3	49.9	51.0	55.8	57.8	55.6
Glo-In-One v2 (ours)	✓	—	88.4	49.9	49.4	71.1	70.0	65.8
Glo-In-One v2 (ours)	✓	✓	**89.7**	**56.7**	**55.7**	**77.6**	**70.2**	**70.0**

H, human training data; R, rodent training data.

Overall, the segmentation performance exhibits a monotonically increasing trend and improved stability across lesion types when rodent data are used as supplementary training data. In addition, the inclusion of a dynamic head further contributes to performance gains by enabling class-specific segmentation. The results demonstrate that incorporating rodent data can effectively enhance the model’s segmentation performance on human glomeruli.

## 5 Discussion

As shown in Fig. 1, it is apparent that some classes in our glomeruli dataset are not completely mutually exclusive but exhibit relationships such as overlap, subset, or superset. For example, in tissue classes, the Bowman’s capsule region of a glomerulus can contain the tuft region, and the tuft region can further contain the mesangium. Another case is the lesion class of global sclerosis, where the mask region covers almost the entire Bowman’s capsule with significant overlap. Traditional multi-head models struggle with these relationships because they tend to segment the image into separate channels, lacking strong associations among them. These associations have a notable impact on the performance of the involved classes.

As shown in Table 3, our proposed model achieves an average improvement of 20% in Dice score for holistic intraglomerular tissue and lesion segmentation using a single dynamic-head network. Notably, the segmentation performance for tissue classes reaches ∼90%, which is substantially higher than that of most lesion classes. In contrast, baseline multi-head models show lower performance on both tissue and lesion segmentation. Lesion accuracy is particularly poor, as most lesion classes fail to exceed a 60% Dice score.

These suggest that glomerular lesions are inherently more challenging to segment compared with tissue structures. Moreover, the poor performance of baseline models indicates their difficulty in handling complex label relationships, especially when there are overlapping or hierarchical dependencies—such as subset or superset structures—between tissue and lesion classes. Our single dynamic-head design addresses these challenges more effectively by modeling shared contextual information across interrelated classes, leading to improved segmentation accuracy.

Table 4 shows that for the rodent-to-human glomerular lesion transfer learning task (R&H2H), our dynamic-head model under hybrid training improves the Dice score by 7% over zero-shot transfer and by 3% over human-only training. This improvement is particularly evident in lesion classes with limited human annotations, such as HS, MA, and SS, where the inclusion of rodent data leads to additional Dice score gains of 8%, 5%, and 1%, respectively. Furthermore, even with the inclusion of additional tissue data (R&H2H+T), the lesion segmentation performance remains stable. In contrast, baseline models exhibit limited improvement when rodent data are introduced. For instance, in some classes such as GS, performance degrades noticeably. In addition, when tissue labels are added, baseline models show significant drops in lesion segmentation performance.

The observations from Table 4 highlight the advantages of our dynamic-head model in leveraging auxiliary rodent data to enhance human lesion segmentation, particularly in data-scarce settings. The limited or even negative impact observed in baseline models may be due to the relatively sufficient amount of human training data in this task, where the inclusion of rodent data introduces domain noise rather than complementary information. Moreover, the considerable performance degradation observed in baseline models upon adding additional tissue classes suggests their susceptibility to confusion caused by label overlap or hierarchical relationships. In contrast, our model demonstrates robustness across varying transfer configurations and label complexities, consistently outperforming all baseline methods in terms of average Dice score.

The results demonstrate the feasibility of adapting such learning strategies from rodent data to human glomerular segmentation using a single network architecture. However, several limitations and potential improvements for our study remain. First, the number of glomerular tissue and lesion classes included in the current dataset is still limited, which may restrict the model’s ability to generalize to rare or unseen pathological patterns. Second, despite the demonstrated benefit of incorporating rodent data through transfer learning, the current approach still relies on a substantial amount of annotated human data to achieve high performance, highlighting the need for further improvements in cross-domain generalization and low-resource adaptation.

Although our model currently relies solely on raw image data for end-to-end learning, a promising future direction would be incorporating additional image-derived morphological features. Specifically, features such as lesion widths, heights, aspect ratios, surface area, or contour-based metrics (e.g., circularity, compactness, and edge sharpness) obtained from segmentation could provide complementary structural context not directly captured by intensity values alone. Integrating these features into the model, either by concatenating them with latent representations or using multi-branch architectures, could help bridge the gap between data-driven learning and expert-guided interpretation. Such a hybrid approach may be particularly beneficial in cases where visual patterns are subtle, heterogeneous, or affected by artifacts.

## 6 Conclusion

In this work, we present and publicly release Glo-In-One-v2, an open-source, containerized toolkit for the holistic identification of glomerular cells, tissues, and lesions in both human and mouse histopathology. Unlike previous tools that target a single species or limited set of structures, Glo-In-One-v2 introduced a single dynamic-head neural network that is more effective for handling multi-class segmentation tasks across species domains. It also expands the spectrum of tissue and lesion classes addressed in glomerular image analysis. The containerized toolkit processes renal WSIs in a fully automated and user-friendly manner—requiring only a single command line to execute—making it accessible to both technical users and clinical researchers. It is trained on a carefully curated dataset comprising five glomerular tissue classes and nine lesion classes from both human and rodent sources. Experimental results demonstrate that Glo-In-One-v2 consistently outperforms multi-head baseline models, particularly in segmenting complex intraglomerular structures characterized by overlapping and hierarchical relationships. Furthermore, the results validate the feasibility and significance of hybrid transfer learning from rodent to human data.

## 7 Appendix: Rodent-to-Rodent Supervised Learning

In transfer learning, ensuring that a model performs well on the source domain is essential, as this capacity forms the foundation for successful adaptation to a target domain. For our study, the model’s proficiency in identifying rodent lesions was evaluated rigorously to validate its predictive robustness for applications in human lesion analysis. Specifically, we trained our models exclusively on rodent domain data, which allowed us to carefully assess their effectiveness in distinguishing intricate lesion patterns. Table 6 present the performance of different models trained by rodent lesion data for prediction on rodent glomerular lesion segmentation. Experimental results indicate that our model consistently outperforms all baseline methods on the rodent dataset, suggesting a higher level of feature extraction and prediction capabilities, establishing a critical foundation for effective transfer learning from rodent to human lesion identification.

**Table 6:** Performance of different models trained by rodent lesion data for prediction on rodent glomerular lesion segmentation. Dice similarity coefficient (%; the higher, the better) is used for evaluation. The bold mark indicates the best performance.

Method	Rodent glomerular lesion								Average
	AH	CD	GS	HS	ML	MA	NS	SS	
U-Nets^25^	49.7	64.7	81.5	70.5	49.4	71.6	62.3	47.4	62.1
DeepLabV3^27^	54.5	66.6	87.9	68.5	52.0	69.5	70.1	66.0	66.9
Multi-class^26^	49.7	49.6	87.6	49.9	49.4	49.4	49.2	47.4	54.0
Swin UNETR^29^	50.3	63.3	84.3	73.3-	50.8	70.5	66.8	49.3	63.6
Segmenter^28^	49.7	51.4	79.8	50.8	50.2	66.8	56.5	51.5	57.1
Ours	**58.7**	**68.1**	**89.7**	**75.5**	**57.2**	**76.9**	**77.9**	**77.7**	**72.7**

## Data Availability

The code used in this study is publicly available at https://github.com/hrlblab/Glo-In-One_v2. However, a portion of the data used in this research includes datasets obtained from Vanderbilt University Medical Center (VUMC). Due to privacy and institutional policies, data sharing requires permission from VUMC and a data use agreement. Therefore, the data cannot be made publicly available at this time. Interested researchers may contact VUMC to explore potential data access options.
